# Mapping the landscape of artificial intelligence in skin cancer research: a bibliometric analysis

**DOI:** 10.3389/fonc.2023.1222426

**Published:** 2023-10-13

**Authors:** Qianwei Liu, Jie Zhang, Yanping Bai

**Affiliations:** ^1^ Graduate School, Beijing University of Chinese Medicine, Beijing, China; ^2^ Library, Chengdu University of Traditional Chinese Medicine, Chengdu, Sichuan, China; ^3^ Department of Dermatology, China-Japan Friendship Hospital, Beijing, China

**Keywords:** skin cancer, artificial intelligence, bibliometric, CiteSpace, VOSviewer

## Abstract

**Objective:**

Artificial intelligence (AI), with its potential to diagnose skin cancer, has the potential to revolutionize future medical and dermatological practices. However, the current knowledge regarding the utilization of AI in skin cancer diagnosis remains somewhat limited, necessitating further research. This study employs visual bibliometric analysis to consolidate and present insights into the evolution and deployment of AI in the context of skin cancer. Through this analysis, we aim to shed light on the research developments, focal areas of interest, and emerging trends within AI and its application to skin cancer diagnosis.

**Methods:**

On July 14, 2023, articles and reviews about the application of AI in skin cancer, spanning the years from 1900 to 2023, were selected from the Web of Science Core Collection. Co-authorship, co-citation, and co-occurrence analyses of countries, institutions, authors, references, and keywords within this field were conducted using a combination of tools, including CiteSpace V (version 6.2. R3), VOSviewer (version 1.6.18), SCImago, Microsoft Excel 2019, and R 4.2.3.

**Results:**

A total of 512 papers matching the search terms and inclusion/exclusion criteria were published between 1991 and 2023. The United States leads in publications with 149, followed by India with 61. Germany holds eight positions among the top 10 institutions, while the United States has two. The most prevalent journals cited were *Cancer*, the *European Journal of Cancer*, and *Sensors*. The most frequently cited keywords include “skin cancer”, “classification”, “artificial intelligence”, and “deep learning”.

**Conclusions:**

Research into the application of AI in skin cancer is rapidly expanding, and an increasing number of scholars are dedicating their efforts to this field. With the advancement of AI technology, new opportunities have arisen to enhance the accuracy of skin imaging diagnosis, treatment based on big data, and prognosis prediction. However, at present, the majority of AI research in the field of skin cancer diagnosis is still in the feasibility study stage. It has not yet made significant progress toward practical implementation in clinical settings. To make substantial strides in this field, there is a need to enhance collaboration between countries and institutions. Despite the potential benefits of AI in skin cancer research, numerous challenges remain to be addressed, including developing robust algorithms, resolving data quality issues, and enhancing results interpretability. Consequently, sustained efforts are essential to surmount these obstacles and facilitate the practical application of AI in skin cancer research.

## 1 Introduction

Artificial intelligence (AI) entails the development of computer systems capable of performing tasks typically requiring human intelligence, such as learning, decision-making, perception, and reasoning. AI systems powered by complex algorithms can execute tasks with human-like intelligence and analyze extensive datasets, rendering AI a promising healthcare research realm (1). Its potential to revolutionize medical practice has garnered significant interest, particularly in medical diagnosis, medical statistics, robotics, and human biology (2). The impact of AI on medicine is particularly pronounced in medical imaging, where widely employed deep-learning methods, such as artificial neural networks (ANNs), play a pivotal (3). Numerous studies have showcased AI’s remarkable capacity to diagnose and treat various clinical diseases by analyzing medical images (4). With its immense potential to transform healthcare, AI stands ready to become a formidable tool for medical professionals.

Skin cancer poses a significant global public health challenge, ranking among the most prevalent cancers. With a rising incidence and substantial morbidity, skin cancer has increasingly captured the attention of the medical and scientific communities. According to Global Cancer Statistics in 2020, skin cancer accounted for 1,198,073 new cases annually, constituting 6.2% of all new cancer cases (5). It is most prevalent among Caucasians due to their lighter skin tones and lower melanin content, which provides less protection against the harmful effects of ultraviolet radiation, consequently elevating their susceptibility to cancer (6). High-confidence data indicate that, in the future, environmental factors and an aging population will further increase skin cancer prevalence worldwide. The incidence rate in Europe and the United States is projected to rise to 40–50 cases per 100,000 individuals per year in the next few decades (7). Two common types of skin cancer are melanoma skin cancer and non-melanoma skin cancer (NMSC). Melanoma is the most lethal form of skin cancer, while NMSC encompasses basal cell carcinoma (BCC), squamous cell carcinoma (SCC), and other variants (8). Several risk factors can elevate the likelihood of developing skin cancer, including exposure to ultraviolet radiation (9–11), a family history of the disease (12), and immunosuppression (13–15). Skin cancer originates from the abnormal proliferation of skin cells. Broadly categorized into melanoma and non-melanoma types, it encompasses malignant melanoma, SCC, and BCC. Although non-melanoma skin cancers are typically surgically curable and non-lethal, dermatologists occasionally face challenges distinguishing between benign and malignant melanoma, posing a significant clinical diagnosis challenge (16). In recent years, the application of AI in dermatology has garnered growing attention and demonstrated substantial potential for enhancing the precision and efficiency of skin cancer diagnosis (17, 18). Several AI-based systems, including deep convolutional neural networks (CNNs) (19), support vector machines, and random forest classifiers (20), have achieved remarkable diagnostic accuracy and can assist dermatologists in their clinical practice.

The field of AI in dermatology is rapidly evolving, with new research regularly published. Without a comprehensive understanding of the latest developments and research trends, researchers may overlook critical findings that could shape their work. Bibliometric analysis provides a powerful tool for scrutinizing the vast literature in skin cancer research and pinpointing essential research hotspots. By examining publication patterns, citation trends, and research collaborations, bibliometric studies offer valuable insights into the current state of research and guide future studies. Furthermore, bibliometric analysis helps identify knowledge gaps, highlighting areas requiring further investigation and informing decision-making in clinical practice (21). Overall, it is a crucial tool for staying updated on the advances in AI research in dermatology and advancing our comprehension of this critical field.

In this study, we conducted bibliometric analysis to identify the countries, institutions, authors, and journals most highly cited and published in the field of AI for skin cancer. We collected literature data from various databases, focusing on the challenges of clinically translating AI in skin cancer. The primary objective of this study was to offer a comprehensive overview of AI’s application and progress in skin cancer research from 1900 to the present. Through bibliometric analysis, we aimed to pinpoint current research advancements, research hotspots, and emerging trends in the field of AI for skin cancer. The findings of this study hold immense value for new researchers, providing insights into the current state of skin cancer research and highlighting areas warranting further investigation.

## 2 Materials and methods

### 2.1 Paper selection

**Figure 1:**
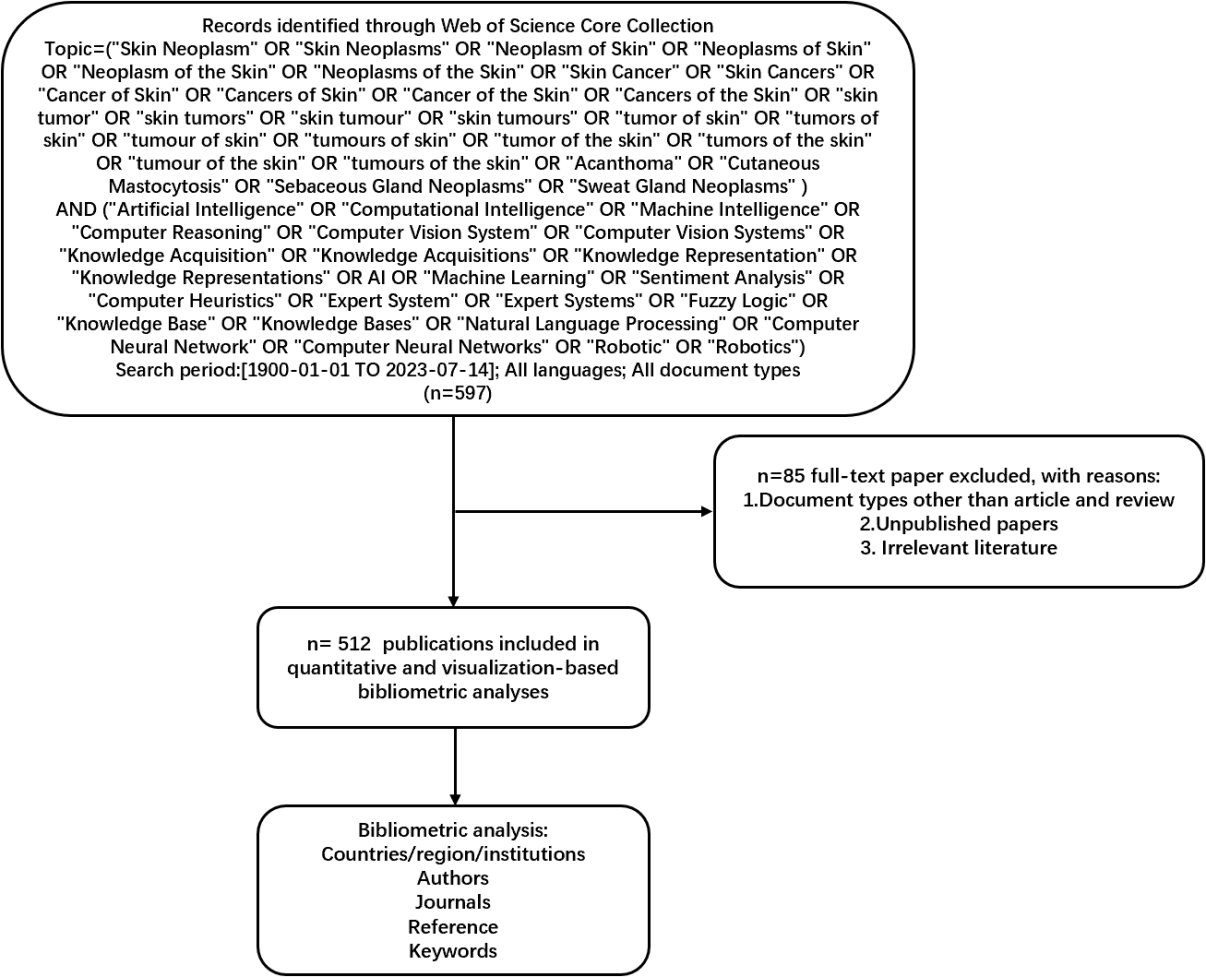
Frame flow diagram for the detailed selection criteria and bibliometric analysis steps of applying AI to the study of skin cancer in the Web of Science Core Collection database. AI, artificial intelligence.

On July 14, 2023, we retrieved all citation data published between January 1, 1900, and July 14, 2023, from the Web of Science Core Collection (WoSCC). We selected WoSCC because it serves as a comprehensive and standardized collection of databases widely utilized in academic circles (22). It encompasses a broader range of research fields than PubMed and spans research from 1900 to the present. This analysis focuses on articles published from 1900 to 2023 in peer-reviewed journals. The detailed search string can be found in **Figure 1**. The document type included articles and reviews. We gathered essential data for each publication, including the title, abstract, authors, institution, country or region, journal, keywords, and references.

### 2.2 Data exclusion

Unpublished documents and document types other than articles were excluded. The citation data were downloaded on July 14, 2023, and some 2022 documents included by WoSCC were not published. Thus, they were excluded from this study. Data with document types other than articles, such as procedures, papers, review articles, meeting abstracts, early access, editorial materials, book chapters, letters, corrections, data papers, books, and retracted publications, were also excluded. **Figure 1** presents the detailed search, exclusion, and analysis processes.

### 2.3 Data analysis

This study employed bibliometric analysis to explore global research trends in the application of AI in skin cancer. Collaborative networks among countries, institutions, journals, keywords, and research categories were analyzed and visualized using CiteSpace (version 6.2. R3), VOSviewer (version 1.6.18), SCImago, Microsoft Excel 2019, and R 4.2.3. The study collected citation data, encompassing the yearly publication count, countries, institutes, journals, and keywords.

## 3 Results

In this section, to understand the current research status and elucidate the broader research landscape, we analyzed annual publication counts, citation trends, publication research categories, and journal preferences. To contextualize the evolution and identify pivotal research themes, we explore the primary contributing authors, countries or regions, and institutions, including their collaborative relationships. We conducted a frequency analysis of critical terms and dynamic keyword analyses to pinpoint research hotspots and reveal future development trends.

### 3.1 Article distribution by publication year

**Figure 2:**
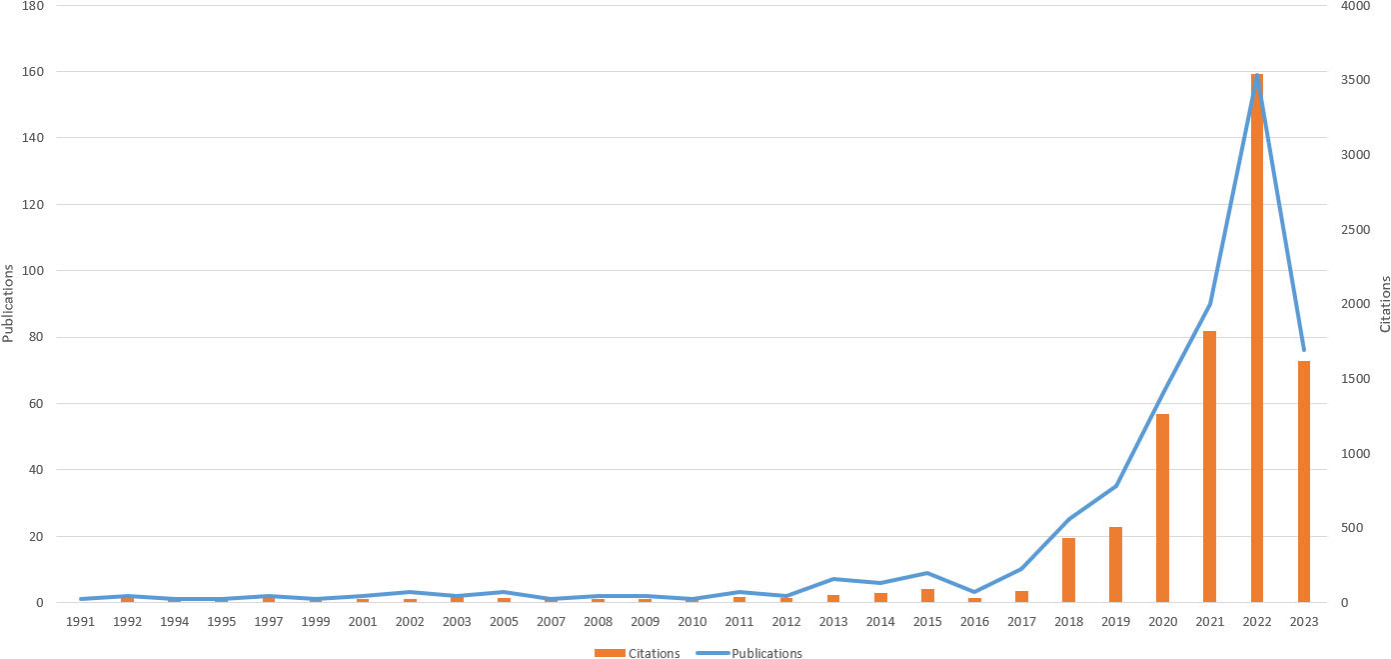
Trends in the number of publications on applying artificial intelligence to the study from 1991 to 2023.

The literature retrieval revealed that research on AI in this topic commenced in 1991. Between 1991 and 2023, 512 papers have been published, allowing us to identify publication trends related to AI in skin cancer research (see **Figure 2**). Studies in this area have been steadily increasing, signifying the establishment of a significant research trend. The annual number of publications in this field has been on the rise, particularly after 2016, experiencing a rapid surge, reaching 159 publications in 2020. This suggests that this research field has garnered increasing attention from researchers in recent years, emerging as a focal point.

### 3.2 Analysis of authors and co-cited authors

A total of 2,539 authors and 13,055 co-cited authors were included in the study. **Table 1** lists the top 10 most productive authors, with Brinker, Titus J, Hekler Achim, and Schadendorf Dirk ranking as the top 3 authors, with 20, 17, and 16 articles, respectively.

**Table 1:** The 10 most productive authors of publications researching the use of AI in skin cancer.

Rank	Author	Research directions	Publications	Citations	H-index	Average citation/publication
1	Brinker, Titus J	Translational oncology; machine learning; cancer prevention	20	614	31	43.86
2	Hekler, Achim	Deep learning; uncertainty; robustness	17	582	25	52.91
3	Schadendorf, Dirk	Cancer; melanoma; translational research; dermatology	16	563	167	56.30
4	Hauschild, Axel	Internal medicine; melanoma; oncology	15	564	102	60.89
5	Haferkamp, Sebastian	Dermatology; oncology; cell biology; experimental medicine; biochemistry; molecular biology	15	553	29	61.44
6	Berking, Carola	Oncology; dermatology; immunology; experimental medicine; biochemistry; molecular biology	14	566	45	62.67
7	Froehling, Stefan	Hematology; oncology; cancer; biology	13	337	76	59.78
8	von kalle, Christof	Oncology; hematology; gene therapy	13	548	104	62.89
9	Utikal, Jochen S	Melanoma; stem cells; skin cancer; dermatology	13	538	75	42.13
10	Schilling, Bastian	Melanoma; skin cancer; immunotherapy; tumor immunology	12	539	55	67.38

AI, artificial intelligence.

*The H-index is a measure of research output and contribution that shows the importance and general impact of the research contributions.

**Figure 3:**
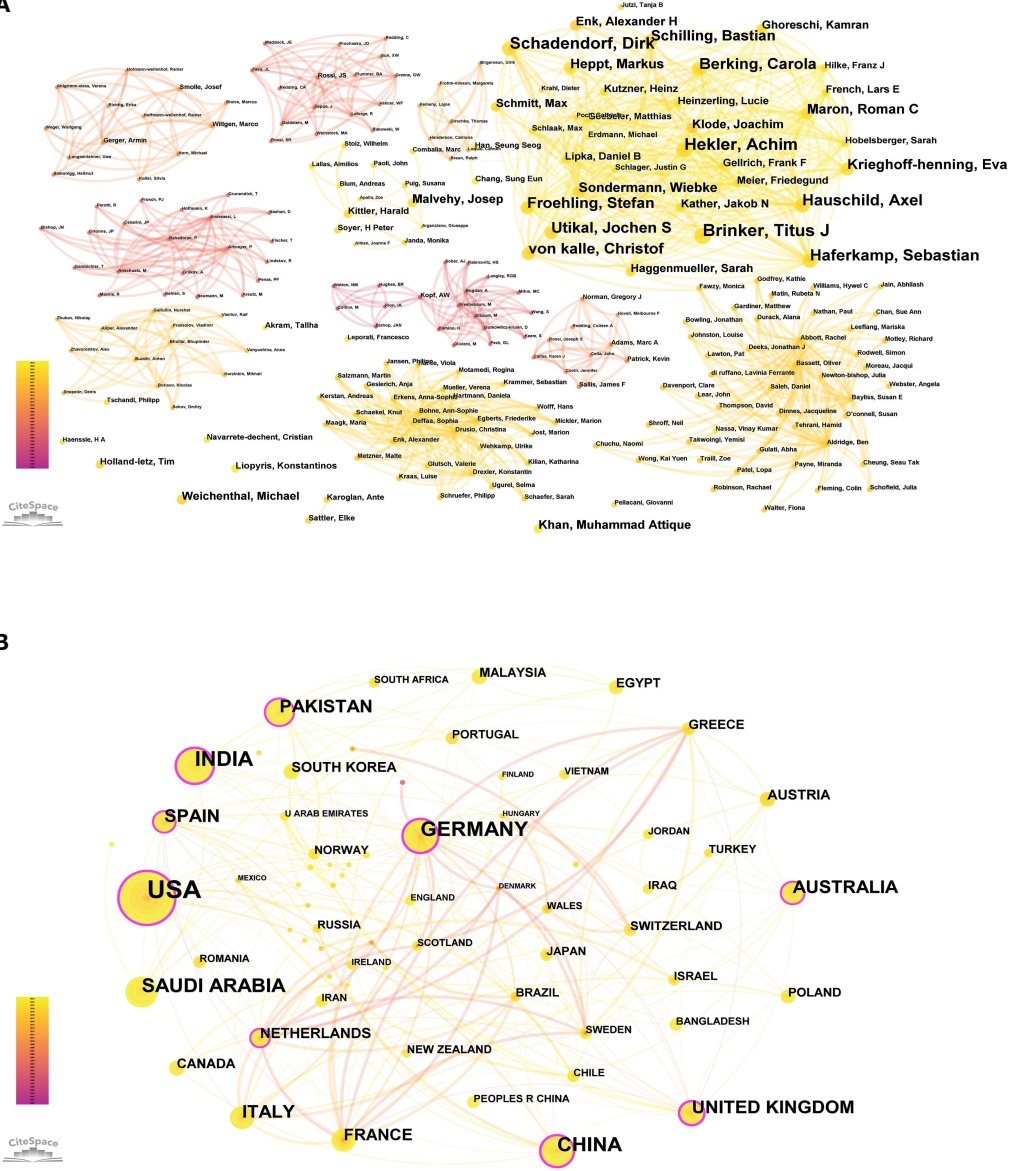
**(A)** Co-occurrence map of authors’ cooperation network. **(B)** The cooperation of countries/regions contributed to publications.

According to Price’s law, the minimum number of papers that core authors should publish in a specific field is represented by N, where N = 0.749 × ηmax 1/2. Here, ηmax is the number of publications by the most prolific author. VOSviewer statistics indicate that ηmax = 20, leading to an approximate value of 3. Hence, authors who have published over three papers are considered core authors. A total of 73 core authors have collectively contributed to 307 papers, meeting the 50% standard proposed by Price. Consequently, it can be inferred that a relatively stable cooperative group of authors has coalesced within this field. CiteSpace was employed to visualize the co-authorship map of authors (see **Figure 2**), and the co-occurrence map of authors’ cooperation network is presented in **Figure 3A**.

### 3.3 Countries or regions and institutes

We identified 70 countries or regions with published studies on skin cancer; an overview of the collaboration among these entities is presented in **Figure 3B**, and the top 10 countries are detailed in **Table 2**. The United States leads in the number of studies published (149), followed by India (61), Germany (57), and China (48). **Figure 3B** highlights the intricate and robust collaborative relationships among different countries. We employed the CiteSpace platform to assess the centrality of countries. As indicated by the color gradient, the bold yellow line in **Figure 3B** represents the most recent partnerships between countries. The size of each circle corresponds to its centrality, with larger circles indicating higher centrality values. The various colored lines connecting the nodes represent collaborations in different years between countries or institutions. The United States (centrality = 0.33), Germany (centrality = 0.25), and China (centrality = 0.21) exhibit extensive collaboration with other countries.

**Table 2:** Top 10 countries/regions.

Rank	Country	Publications	Citations	Centrality	Average citation/publication
1	USA	149	8587	0.33	57.63
2	India	61	472	0.21	7.74
3	Germany	57	1176	0.25	20.63
4	China	48	360	0.23	7.50
5	Saudi Arabia	44	287	0.09	6.52
6	Pakistan	32	762	0.16	23.81
7	Italy	32	729	0.03	22.78
8	UK	32	246	0.21	7.69
9	Spain	30	759	0.19	25.30
10	Australia	30	699	0.12	23.30

**Figure 4:**
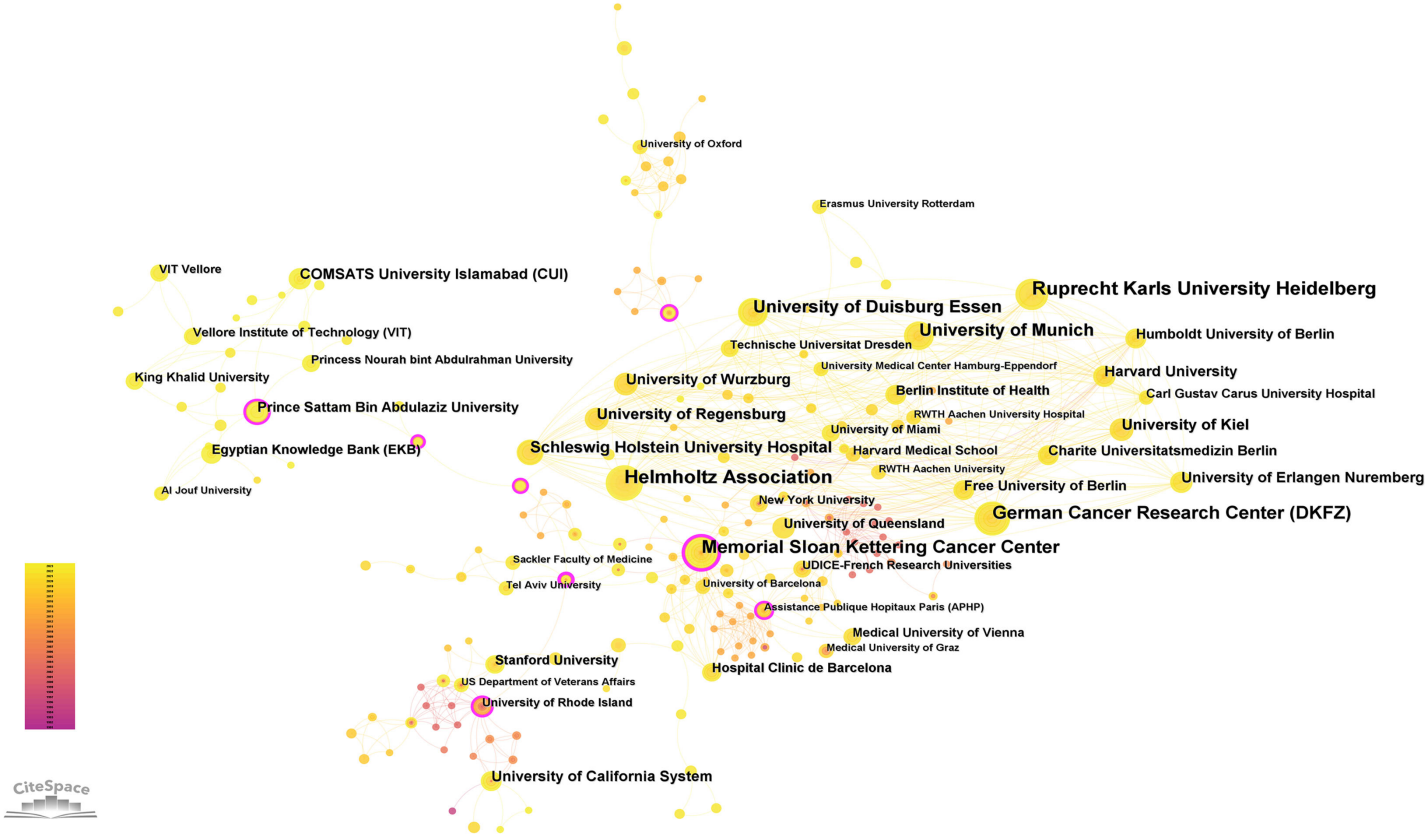
The cooperation of institutions contributed to publications.

We identified 1,127 institutions contributing to this field, with the top 10 listed in **Table 3**. Ruprecht Karls University Heidelberg (26) leads, followed by the Helmholtz Association (25), the German Cancer Research Center (DKFZ) (24), and the Memorial Sloan Kettering Cancer Center (22). Data analysis revealed that eight of the top 10 institutions are located in Germany, while the remaining two are in the United States. **Figure 4** illustrates the cooperative relationships among institutions, indicating that the collaboration is both widespread and partly concentrated.

**Table 3:** Top 10 relevant institutions.

Rank	Institution	Countries	Publications	Centrality
1	Ruprecht Karls University Heidelberg	Germany	26	0
2	Helmholtz Association	Germany	25	0.09
3	German Cancer Research Center (DKFZ)	Germany	24	0.1
4	Memorial Sloan Kettering Cancer Center	USA	22	0.38
5	University of Duisburg Essen	Germany	20	0.02
6	University of Munich	Germany	19	0.06
7	Schleswig Holstein University Hospital	Germany	15	0
8	University of Regensburg	Germany	13	0
9	University of Kiel	Germany	13	0
10	Harvard University	USA	12	0.03

It is evident that there is a need to strengthen both intra-regional and international exchanges and cooperation. The findings indicate that medical colleges and hospitals constituted a relatively significant proportion within the research domain, serving as the primary drivers of research and publication. Inter-institutional collaboration is predominantly confined to local regions, with limited cross-regional cooperation observed. These results underscore the importance of future teams avoiding singular institutional or regional focus and, instead, emphasizing enhanced inter-team collaboration.

### 3.4 Journals

In any research field, the referential relationships among academic journals typically reflect knowledge exchange, citing studies representing knowledge frontiers and referenced studies forming the knowledge foundation. **Table 4** displays the top 10 citing journals, with the most frequently cited journals in the included references being *Cancer* (21 citations), *Diagnostics* (19 citations), and the *European Journal of Cancer* (17 citations).

**Table 4:** Top 10 citing references on the application of AI in skin cancer.

Rank	Journal	Publications	IF (2021)	JCR (2021)	Citations	H-index	Average citation/publication
1	*Cancer*	21	6.921	Q1	239	111	11.38
2	*Diagnostics*	19	3.992	Q2	60	52	3.16
3	*European Journal of Cancer*	17	10.002	Q2	793	235	46.65
4	*Sensors*	16	3.847	Q2	87	219	5.44
5	*Computers in Biology and Medicine*	11	6.698	Q1	105	113	9.55
6	*Multimedia Tools and Applications*	11	2.577	Q2	46	93	4.18
7	*Journal of the American Academy of Dermatology*	9	15.487	Q1	460	229	51.11
8	*IEEE Access*	8	3.476	Q2	104	204	13.00
9	*Journal of Investigative Dermatology*	8	7.59	Q1	159	220	19.88
10	*Journal of the European Academy of Dermatology and Venereology*	8	9.228	Q1	117	123	14.63

AI, artificial intelligence; IF, impact factor; JCR, Journal Citation Report.

**Figure 5:**
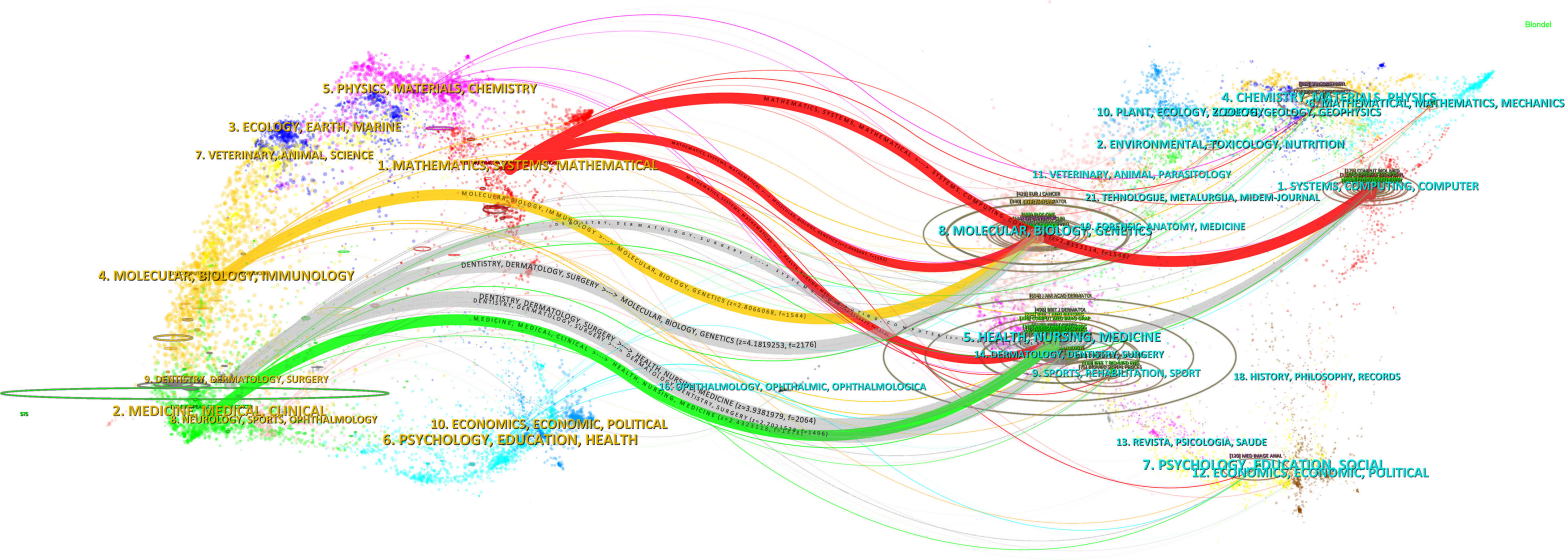
Dual-map overlay of journals contributing to publications.

Dual-map overlay visualization can illustrate the distribution of papers in each subject, citation trajectory, the center of gravity shifts, and other relevant information (23). **Figure 5** presents a dual-map overlay of journals, showcasing citing and cited journals on the left and right, respectively, with citation relationships represented by colored paths.

There were primarily four citation paths, with citing papers mainly concentrated in four fields: 1) molecular biology and immunology; 2) medicine, medical, and clinical disciplines; 3) mathematics, systems, and mathematical studies; and 4) dentistry, dermatology, and surgery. The cited papers were mainly situated in four fields: 1) systems, computing, and computer science; 2) molecular biology and genetics; 3) health, nursing, and medicine; and 4) dermatology, dentistry, and surgery.

### 3.5 Keywords

In a research field, a hot topic is characterized by a high frequency of keywords. In contrast, high-centrality keywords indicate the location and significance of associated research content within that field. To conduct a keyword analysis of global research trends in the application of AI in skin cancer, we retrieved and examined relevant literature to identify the most frequently used keywords.

The top 10 keywords are presented in **Table 5**, which can be categorized into three topics for further discussion. i) Research subject: “skin cancer” and “melanoma” have the highest frequency of occurrence. This is attributed to melanoma having the highest fatality rate and is one of the most aggressive and lethal forms of cancer, with increasing worldwide incidence in recent decades (24). ii) Research purpose: the keyword “diagnosis”, which first appeared in 1992, reflects the primary objective of applying AI to skin cancer. Meanwhile, the emergence of “classification” and “segmentation” since 2003 indicates more refined objectives in the diagnostic process. The high frequency of these keywords is closely related to developing relevant image-processing algorithms. iii) Research techniques: “machine learning” first appeared in 2003. Machine learning is a significant branch of AI, and strictly speaking, both “deep learning” and “CNN” fall under the umbrella of machine learning algorithms (25). Interestingly, we observed that “neural networks” appeared in related literature as early as 1994, while “deep learning” and “CNN”, derived from this concept, only began to frequently appear decades later, in 2016 and 2019, respectively. This aligns with the trajectory of AI development, where recent innovations in computer hardware technology have revitalized past theories. This insight suggests that future breakthroughs in computer hardware technology may play a critical role in advancing AI for clinical applications in skin cancer diagnosis. However, future research on more generalized AI algorithms applicable to various types of skin cancer diagnosis may hold even greater value.

**Table 5:** The top 10 keywords on the application of AI in skin cancer.

Rank	Keywords	Occurrence	Centrality	Year of first appearance
1	Skin cancer	189	0.19	1992
2	Classification	145	0.11	2003
3	Artificial intelligence	132	0.03	1992
4	Deep learning	126	0.06	2016
5	Machine learning	121	0.05	2003
6	Diagnosis	110	0.09	1992
7	Melanoma	104	0.13	1992
8	CNN	54	0.03	2019
9	Cancer	50	0.13	1997
10	Segmentation	49	0.04	2003

AI, artificial intelligence; CNN, convolutional neural network.

**Figure 6:**
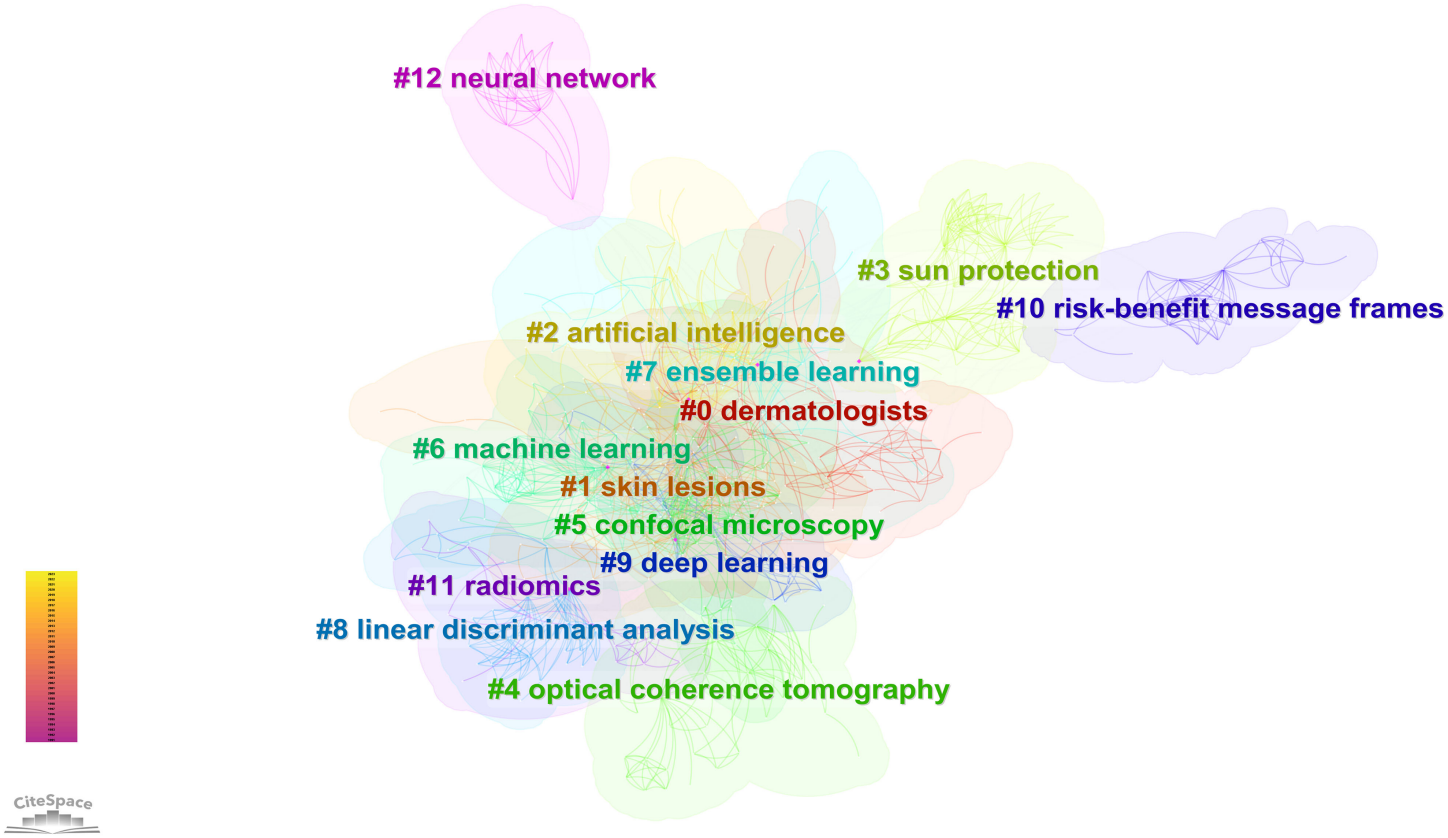
Clustering map of keywords.

**Figure 7:**
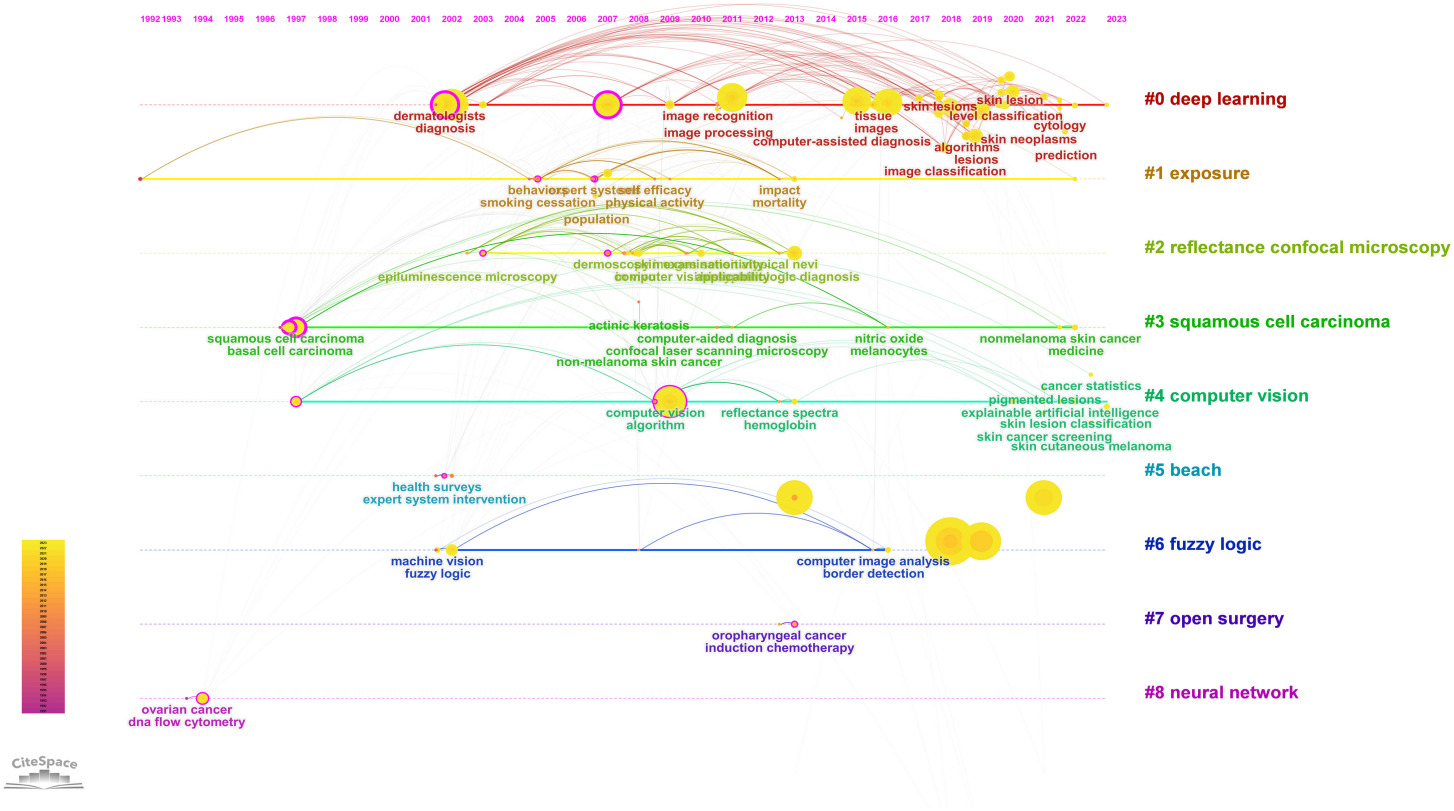
Visualization map of keyword analysis timeline viewer.

Keyword clusters can illustrate the structural system of related research fields. The results of keywords clustering revealed Q = 0.6142 > 0.3 and S = 0.8309 > 0.7 in **Figure 6**, indicating the significance of the cluster structure and the credibility of the results (26). The timeline map visualizes the number of keywords within each cluster. A cluster’s significance is determined by the number of keywords it contains, and it also reveals the temporal span of keywords within each cluster. This facilitates the examination of the emergence, growth, and decline of specific research clusters, thereby assisting in exploring temporal patterns characterizing the research field represented by these clusters (27). We obtained the timeline view for the 13 clusters in **Figure 7**, where keywords from the same cluster are placed on the same horizontal line. The chronological occurrence of keywords is positioned at the top of the view, with the timing progressing toward the right. “#2 Artificial intelligence” first appeared in 1992 and was the earliest keyword. The research focused on “#7 Radiomics”, which appeared latest among the 13 clusters. The timeline for “#0 Dermatologists”, “#1 Skin lesions”, “#2 Artificial intelligence”, “#4 Optical coherence tomography”, “#7 Ensemble learning”, “#11 Radiomics”, and “#12 Neural network” indicates that they are closest to 2023. This suggests that these subjects have recently gained more attention and are likely to become research hotspots soon.

**Figure 8:**
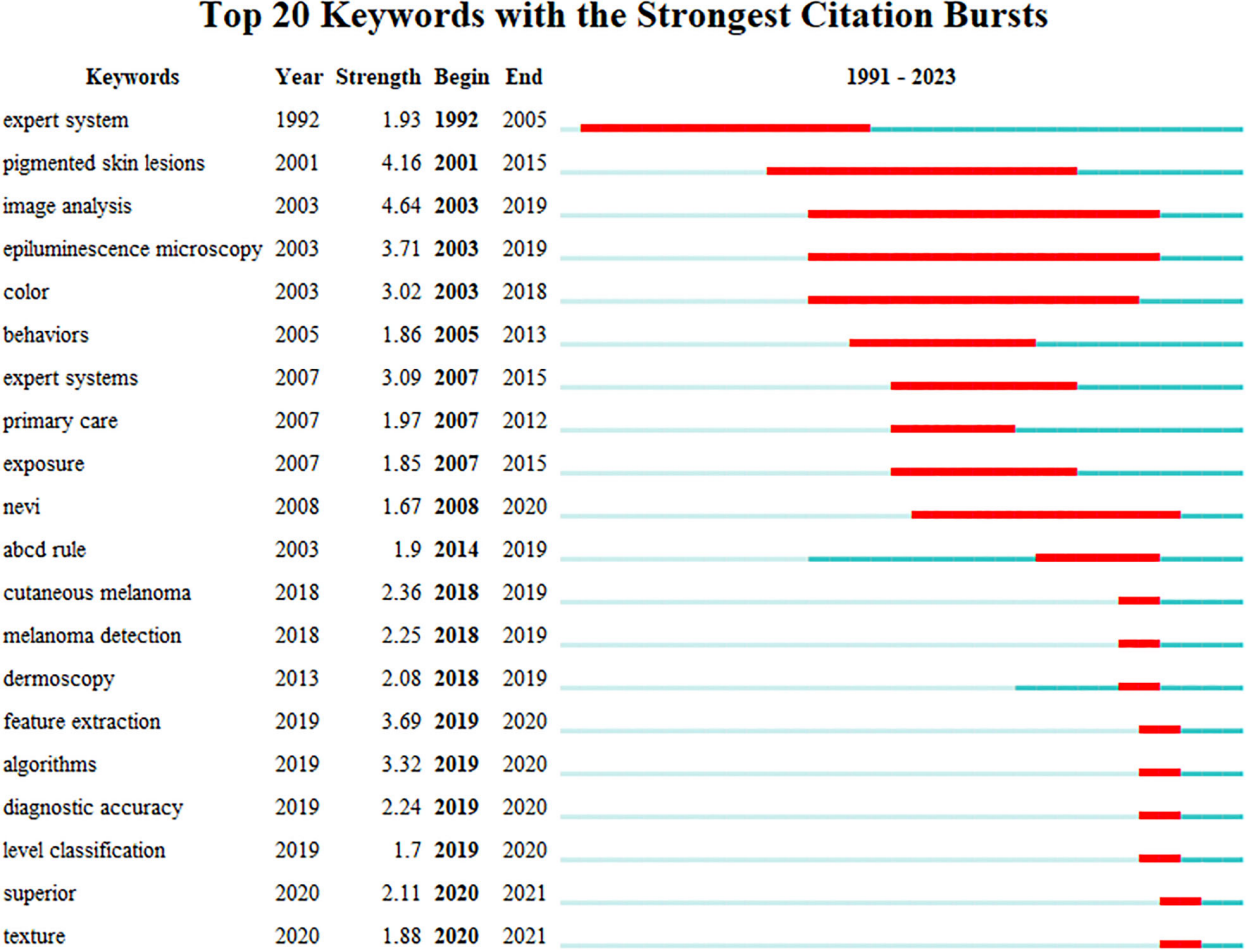
Top 20 keywords with the most robust citation bursts of publications on the application of AI in skin cancer. Red indicates the emergence ofkeywords. AI, artificial intelligence.

The analysis of emerging burst keywords can reflect the changing trends in the field’s hot topics, further supporting the findings of this study. The strength value indicates the frequency of citations. The blue line represents the time interval, while the red line represents the duration of the citation burst. In **Figure 8**, it is evident that “Pigmented skin lesions”, “Image analysis”, and “Epiluminescence microscopy” exhibited the highest outburst intensity among keywords. Additionally, we identified the emergence of new keywords, depicted by the red grid, with “Feature extraction”, “Algorithms”, “diagnostic accuracy”, “level classification”, superior”, and “texture” being particularly popular from 2019 to 2021. Notably, “Image analysis” had the most extended usage from 2003 to 2019. Our keyword analysis offers valuable insights into the most frequently used and emerging keywords in AI application research for skin cancer.

## 4 Discussion

To the best of our knowledge, while bibliometrics has been widely utilized to investigate trends in various research domains (28–30), this study can be considered the inaugural, all-encompassing global mapping and analysis of scientific research about AI in the context of skin cancer.

### 4.1 General data

In this study, 512 papers, meeting the search terms and adhering to the inclusion/exclusion criteria, were published between 1991 and 2023. A corresponding network diagram was constructed for visual analysis, aiming to understand the dynamic evolution process of core researchers, research focal points, and research frontiers in applying AI to skin disease research. This analysis also serves to explore the developmental trends within this field.

i.The consistent year-on-year increase in publications within this field, notably surging after 2016, aligns with the recognition of the open AI ecosystem as one of the 10 most pivotal emerging technologies by the World Economic Forum in the same year. This suggests a significant correlation between these trends (31). There is indeed a correlation between them. The top three authors were Brinker Titus J (20), Hekler Achim (17), and Schadendorf Dirk (16). The United States published the highest number of studies (149), followed by India (61). Germany claims eight positions among the top 10 institutions, while the United States holds two. The most frequently cited journals in the included references were *Cancer* (21 citations), *Diagnostics* (19 citations), and *European Journal of Cancer* (17 citations).

ii.Intra-regional and international exchanges and collaboration must be bolstered. The findings unveiled that medical colleges and hospitals comprised a significant portion of the research domain, acting as the principal driving forces behind research and publication. Inter-institutional collaboration was predominantly confined to local regions, with minimal cross-regional association observed. These results indicate that future teams should endeavor to steer clear of singular institutional or regional focus instead of prioritizing enhanced inter-team collaboration.

### 4.2 Research status of the implementation of AI in skin cancer

The incidence of skin cancer is progressively rising each year. Despite its comparatively low mortality rate, it continues to place a substantial financial burden on health services. It can lead to severe mental health issues, mainly because most skin cancers manifest in highly conspicuous areas of the body. Due to limited awareness of screening, the absence of distinct lesion characteristics in early-stage skin cancer, and inadequate clinical proficiency and services, most patients receive diagnoses at advanced stages, leading to unfavorable prognoses. Hence, there exists an immediate necessity for AI systems to aid healthcare professionals in this field (32).

AI is a branch of computer science that enables machines to perform human-like tasks through learning from experience (33). Machine learning, a subfield of AI, focuses on training computers to enhance their performance in specific tasks through experiential learning (26). In machine learning, algorithms self-train by directly analyzing data to discern patterns rather than composing software with explicit instructions for a particular task. Two common types of machine learning tasks are supervised and unsupervised learning (34, 35). Supervised learning involves algorithms utilizing labeled training, entails data classification, and programming the relationship between input and output data. Algorithms for classification in supervised learning include ANNs, decision tree networks, support vector machines, and Bayesian networks. Unsupervised learning employs algorithms to discern hidden patterns within datasets, yielding diverse outcomes such as cluster analysis, dimension reduction, and association. Deep learning, a subset of machine learning, is rooted in deep neural network architectures. Natural language processing is another machine learning application that employs software programming to comprehend and manipulate natural language text or speech for practical purposes (33). Currently, AI finds extensive utility in skin cancer across various aspects.

#### 4.2.1 Image analysis, disease prediction, and early diagnosis

AI is pivotal in aiding healthcare professionals in achieving early disease diagnosis and prognosis through computer vision technology. The general steps involved in this process encompass image acquisition, preprocessing, segmentation, feature extraction, and lesion classification. For instance, in the case of skin cancer, diagnosis primarily relies on visual examination. This typically initiates with an initial clinical screening, followed potentially by dermoscopic analysis, biopsy, and histopathological examination (36). Medical image analysis, early diagnosis, and disease forecast: AI’s contribution to early diagnosis and disease prediction is facilitated by computer vision technology. The critical steps in this process include image acquisition, preprocessing, segmentation, feature extraction, and lesion classification (37–39). The process commences with acquiring medical images from various modalities, such as high-resolution photographs of skin lesions, optical endoscopy imaging, optical coherence tomography, photoacoustic imaging, diffuse optical tomography, super-resolution microscopy imaging, Raman spectroscopy imaging, and fluorescence imaging. These images serve as the primary data source for the AI system. Since raw medical images often exhibit defects, several image preprocessing steps are employed to enhance image quality, correct distortions, standardize illumination, and reduce noise. Image segmentation is a critical stage in identifying regions of interest in medical images, where the image is divided into segments corresponding to anatomical structures, lesions, or abnormalities. Techniques like thresholding, area growing, edge detection, or advanced deep methods are utilized for accurate segmentation. Extracting relevant information from segmented regions is crucial for AI systems to make informed decisions. Feature extraction quantifies various aspects of the segmented region, including shape, texture, intensity, and spatial relationships. These extracted features provide a concise representation of essential properties. AI systems leverage machine learning or deep-learning algorithms to classify lesions using the extracted features. These algorithms are trained on diverse datasets, enabling them to classify lesions into different disease categories or predict the likelihood of specific medical conditions (40). A recent study conducted a cross-sectional examination using 100 randomly selected dermoscopic images (comprising 50 melanomas, 44 nevi, and 6 lentigines) from an international computer vision melanoma challenge dataset (n = 379). We utilized five non-learned and machine learning methods to combine individual automated predictions into “fusion” algorithms. Dermatologists exhibited an average sensitivity and specificity of 82% and 59%, respectively. At 82% sensitivity, dermatologists’ specificity was comparable to the top-performing challenge algorithm (59% *vs.* 62%, p = .68). Still, it was lower than the best-performing fusion algorithm (59% *vs.* 76%, p = .02). The receiver operating characteristic area of the top fusion algorithm exceeded the mean receiver operating distinct area of dermatologists (0.86 *vs.* 0.71, p = .001) (39). In a comparative study (41), 4,204 biopsy-proven images of melanoma and nevi (1:1) were employed for training a CNN. Innovative deep-learning techniques were incorporated. For the experiment, an additional 804 biopsy-proven dermoscopic images of melanoma and nevi (1:1) were randomly presented to dermatologists. They evaluated the quality of each image and provided their recommended treatment (amounting to 19,296 recommendations in total). The findings revealed that dermatologists achieved a sensitivity of 67.2% and a specificity of 62.2% in lesion classification. In contrast, the trained CNN demonstrated higher sensitivity at 82.3% and specificity at 77.9%.

AI has emerged as the gold standard for diagnosing histopathological conditions, garnering significant attention in the medical field. Manual interpretation of pathological images is a time-consuming and labor-intensive process. AI can identify subtle lesions that may challenge even experienced pathologists. This not only alleviates the workload on pathologists but also enhances the accuracy of disease diagnosis. In the context of Asian extramammary Paget’s disease (EMPD) pathological image screening, a deep-learning method was developed and validated by HU. This method employs five distinct deep CNNs, namely, ResNet34, ResNet50, MobileNetV2, GoogLeNet, and VGG16. Its primary objective is to differentiate between Paget’s cells and normal cells. A retrospective single-center study demonstrated that the proposed method yields quantitative, rapid, and consistent results. The ResNet34 model outperformed the other, achieving an impressive accuracy rate of 95.522% when analyzing pathological images captured at a magnification of ×40. This innovation can significantly enhance grassroots pathologists’ efficiency and accuracy, ultimately improving patient care (42).

#### 4.2.2 Therapeutic response prediction

In a retrospective study, 79 patients diagnosed with scalp cutaneous squamous cell carcinoma (cSCC) underwent radiotherapy, among whom 22 had previously undergone surgical procedures. Following radiotherapy, 66 patients exhibited partial responses, six demonstrated no response, and five were lost to follow-up. By employing machine learning techniques, specifically an ANN, unique attributes characterizing radiation-responsive cSCC patients were discerned. These included age-specific T2-stage cSCC and distinct lesion types, culminating in the development of predictive models exhibiting remarkable sensitivity (85.7%), specificity (97.6%), and an impressive overall accuracy rate of 91.7% (21).

In situations where multidisciplinary teams may not be readily available, AI-based algorithms can assume a pivotal role in offering guidance for patient care. Supervised machine learning algorithms were harnessed in a specific study to fashion a risk-stratification model. This model notably showcased a 45.1% accuracy in predicting the treatment choices embraced by multidisciplinary medical teams. These choices encompassed a spectrum of options, including conventional treatments, surgical resection, and radiotherapy for addressing intricate BCC cases. Furthermore, it exhibited a 37.5% predictive accuracy in facilitating triage decisions specifically for Mohs micrographic surgery (43).

#### 4.2.3 AI-assisted surgery

Radical resection and amputation represent the optimal approach for averting recurrence and fatal metastasis in cases of malignant skin tumors. Robot-assisted surgical systems can significantly enhance surgical precision and control, making complex surgeries safer and more effective. Sohn (44) applied this technique to two patients afflicted by metastatic melanoma in the pelvic region. The results demonstrate the feasibility and safety of robotic pelvic lymphadenectomy in managing patients grappling with metastatic melanoma involving the pelvic lymph nodes. Compared to the conventional open procedure, pelvic lymphadenectomy with robotic assistance offers superior visualization and minimal morbidity. Kim (45) elucidated the surgical procedures of robot-assisted anterior pelvic exenteration (rAPE) coupled with ileal conduit urinary diversion for vulvovaginal malignant melanoma. Furthermore, Alexis (46) employed robot-assisted inguinal video endoscopic lymphadenectomy to treat a 42-year-old male patient diagnosed with acral lentiginous melanoma and palpable inguinal nodes, classified as T2 N1 M0.

#### 4.2.4 Teledermatology

AI has made significant recent advancements in teledermatology (TD), a progress that the COVID-19 pandemic has notably accelerated. This merging of AI and TD is heralding a transformation in remote dermatological care, potentially enhancing diagnostic accuracy and promoting equitable healthcare access (47). AI’s image analysis and data interpretation capacity empowers healthcare professionals to employ these tools for remote dermatological diagnosis and treatment, ensuring patient care quality and safety.

### 4.3 Research hotspots and frontiers

Research into the application of AI in the diagnosis and management of skin cancer has witnessed rapid growth in recent years, unveiling numerous promising avenues for future investigations. Much of this research has centered on melanoma, a skin cancer with a high mortality rate that has garnered extensive attention. Early screening and diagnosis are pivotal in enhancing patient survival, especially given the time-consuming nature of manual examinations and the potential for confusion with benign skin lesions, even among seasoned dermatologists. Moreover, the disease diagnosis can be particularly challenging in regions with limited medical resources. To address these challenges, expanding skin disease image databases to encompass a more comprehensive array of skin cancer types and other complex-to-diagnose benign conditions may offer valuable clinical support in the future.

Although AI’s role in analyzing skin mirror images and skin pathological images has been extensively discussed, fewer reports on AI research leverage other types of photographic samples. Researchers can delve into additional sample types to tackle factors such as variations in zoom, angle, and lighting conditions, which can significantly impact the accuracy of clinical image analysis.

Moreover, the technological advancements in AI that have been harnessed in the context of other diseases are increasingly finding application in skin cancer. This includes the implementation of personalized and precision medicine approaches that draw from diverse data sources, encompassing genetic databases, medical records, tissue banks, and various clinical databases. The influx of data, often called “big data”, necessitates robust computing power for efficient processing and analysis. AI, coupled with the exponential growth in computing power capabilities, augments our analytical capacity and facilitates the seamless integration of various datasets to extract meaningful insights. Precision medicine relies on extensive population-level data to inform individualized treatment decisions, and AI plays a pivotal role in navigating and interpreting this intricate landscape (48, 49).

Much of the existing research compares dermatologists with AI, but these two entities do not have a hostile relationship. AI does not aim to replace human doctors in making independent diagnoses; instead, it is a valuable tool for assisting doctors in diagnosis and treatment. One study, for instance, investigated the potential benefits of integrating human expertise with artificial intelligence for the classification of skin cancer (50). Dermatologists should not perceive AI as a threat to their professional knowledge; instead, they should consider it a complementary resource in clinical practice for the foreseeable future. Therefore, future research should continue to explore the synergy between humans and computers in the field. By better understanding AI concepts, dermatologists can enhance their ability to provide improved patient care and treatment.

AI faces several challenges in medical applications: 1) complex clinical scenarios characterized by inconsistent disease classification, a lack of standardized diagnostic criteria, and subjective evaluation standards can pose significant hurdles when designing disease diagnosis systems. This complexity may result in variations in the performance of intelligent algorithms (51). 2) The relatively limited sample size available for training AI models, coupled with incomplete and fragmented databases, along with variations among tested individuals, raises legitimate concerns regarding the actual effectiveness of AI systems. 3) Current auxiliary diagnostic systems still lack the capability for independent diagnosis, while the interpretability of intelligent diagnostic systems falls short of the desired standards. Therefore, further validation and confirmation of their efficacy become imperative (52).

To address these challenges and promote the advancement of AI in diagnosis, the following recommendations are proposed: 1) establishing standardized disease classifications and diagnostic criteria: to enhance the development of more robust and practical diagnostic systems, it is imperative to establish standardized disease classifications and diagnostic criteria. This will reduce the reliance on subjective standards, ensuring the generality and reliability of these systems. Active involvement of experienced clinical professionals in the development process is paramount. 2) Curative diverse and extensive datasets: accessing diverse and extensive datasets is essential to refine AI models effectively. Creating a unified and high-quality image database that can be continuously optimized and expanded to meet the diagnostic demands of various diseases is vital. This approach helps overcome the limitations associated with sample size. 3) Strengthening professional training: maximizing the utilization rate of AI technology necessitates strengthening training programs for relevant professionals. Ensuring that healthcare professionals are well-equipped to integrate AI into their practice is crucial for successfully adopting and implementing AI-based diagnostic tools. These recommendations aim to propel the field of AI in diagnosis forward, fostering innovation and improved healthcare outcomes while adhering to rigorous scientific and professional standards.

The application of AI in skin cancer diagnosis and management represents a dynamically evolving field with numerous promising research avenues. By persistently exploring these directions, researchers can forge innovative solutions aimed at enhancing the precision, efficiency, and availability of skin cancer diagnosis, all while conscientiously addressing the ethical and legal dimensions of this undertaking. To establish the foundation for the legal recognition of AI-based diagnosis, researchers should undertake concerted efforts to advance the technology and secure the endorsements for autonomous diagnosis when the technology reaches an adequate level of maturity.

## 5 Limitation

This investigation has certain limitations that warrant acknowledgment. First, the time frame of the search strategy may have led to the exclusion of emerging research hotspots in 2023, as it only encompassed records up to July 14 of that year. Second, this study exclusively concentrates on English literature, potentially overlooking crucial contributions in other languages. Third, the scope of this research did not extend literature from other databases, primarily because of the impracticability of simultaneously combining and analyzing data from multiple databases.

## 6 Conclusion

To summarize, the integration of AI in dermatology is evident, and with the rapid advancement in AI efficiency and the mounting workload of healthcare professionals, incorporating AI into healthcare could become a fundamental element of forward-looking medical practice. This transition will enable healthcare professionals to prioritize the emotional facets of patient care, including fostering empathy and engaging in compassionate interactions, which play a crucial role in nurturing the doctor–patient relationship. The current use of AI in skin cancer remains insufficient, with future efforts aimed at enhancing diagnostic accuracy through deep-learning techniques and predicting treatment outcomes and prognoses based on extensive data analysis. The primary challenges faced in the application of AI in skin cancer encompass personalized data acquisition, data quality assurance, complexity in data processing, reproducibility of AI algorithms, and ensuring the reliability of AI in supporting diagnostic decisions.

## Data availability statement

The raw data supporting the conclusions of this article will be made available by the authors, without undue reservation.

## Author contributions

The study was created by QL. QL and JZ gathered the data and prepared the paper. The data were examined by QL. The manuscript was revised and reviewed by YB. The essay was written by all of the writers. All authors contributed to the article and approved the submitted version.

## References

[1] Bengio Y LeCun Y . Scaling learning algorithms towards AI. Large-Scale Kernel Machines (2007) 34:1–41. doi: 10.7551/mitpress/7496.003.0016

[2] Topol EJ . High-performance medicine: The convergence of human and artificial intelligence. Nat Med (2019) 25:44–56. doi: 10.1038/s41591-018-0300-7 30617339

[3] Jiang F Jiang Y Zhi H Dong Y Li H Ma S . Artificial intelligence in healthcare: Past, present and future. Stroke Vasc Neurol (2017) 2:230–43. doi: 10.1136/svn-2017-000101 PMC5829945 29507784

[4] Ray A Gupta A Al A . Skin lesion classification with deep convolutional neural network: Process development and validation. JMIR Dermatol (2020) 3:e18438. doi: 10.2196/18438

[5] Sung H Ferlay J Siegel RL Laversanne M Soerjomataram I Jemal A . Global cancer statistics 2020: GLOBOCAN estimates of incidence and mortality worldwide for 36 cancers in 185 countries. CA Cancer J Clin CA: C. A. Cancer J Clin (2021) 71:209–49. doi: 10.3322/caac.21660 33538338

[6] Bradford PT . Skin cancer in skin of color. Dermatol Nurs (2009) 21(4):170–177, 206. 19691228 PMC2757062

[7] Apalla Z Lallas A Sotiriou E Lazaridou E Ioannides D . Epidemiological trends in skin cancer. Dermatol Pract Concept (2017) 7:1–6. doi: 10.5826/dpc.0702a01 PMC5424654 28515985

[8] Khayyati Kohnehshahri M Sarkesh A Mohamed Khosroshahi L HajiEsmailPoor Z Aghebati-Maleki A Yousefi M . Current status of skin cancers with a focus on immunology and immunotherapy. Cancer Cell Int (2023) 23:174. doi: 10.1186/s12935-023-03012-7 37605149 PMC10440946

[9] Paulo MS Symanzik C Ádam B Gobba F Kezic S van der Molen HF Peters CE Rocholl M Tenkate T John SM Loney T Modenese A Wittlich M . Risk of cutaneous squamous cell carcinoma due to occupational exposure to solar ultraviolet radiation: Protocol for a systematic review and meta-analysis. PLoS One (2023) 18(3):e0282664. 36867594 10.1371/journal.pone.0282664 PMC9983864

[10] Leiter U Keim U Garbe C . Epidemiology of skin cancer: Update 2019. Adv Exp Med Biol (2020) 1268:123–39. doi: 10.1007/978-3-030-46227-7_6 32918216

[11] Narayanan DL Saladi RN Fox JL . Ultraviolet radiation and skin cancer. Int J Dermatol (2010) 49(9):978–86. doi: 10.1111/j.1365-4632.2010.04474.x 20883261

[12] Lichtenberger BM Gerber PA Holcmann M Buhren BA Amberg N Smolle V . Epidermal EGFR controls cutaneous host defense and prevents inflammation. Sci Transl Med (2013) 5(199):199ra111. doi: 10.1126/scitranslmed.3005886 23966300

[13] Collins L Quinn A Stasko T . Skin cancer and immunosuppression. Dermatol Clin (2019) 37(1):83–94. doi: 10.1016/j.det.2018.07.009 30466691

[14] Khayyati Kohnehshahri M Sarkesh A Mohamed Khosroshahi L HajiEsmailPoor Z Aghebati-Maleki A Yousefi M . Current status of skin cancers with a focus on immunology and immunotherapy. Cancer Cell Int (2023) 23(1):174. doi: 10.1186/s12935-023-03012-7 37605149 PMC10440946

[15] Halliday GM Lyons JG . Inflammatory doses of UV may not be necessary for skin carcinogenesis. Photochem Photobiol (2008) 84(2):272–83. doi: 10.1111/j.1751-1097.2007.00247.x 18353168

[16] Samarasinghe V Madan V . Nonmelanoma skin cancer. J Cutan Aesthet Surg (2012) 5:3–10. doi: 10.4103/0974-2077.94323 22557848 PMC3339125

[17] Diab AG Fayez N El-Seddek MM . Accurate skin cancer diagnosis based on convolutional neural networks. IJEECS (2022) 25:1429–41. doi: 10.11591/ijeecs.v25.i3.pp1429-1441

[18] Brinker TJ Hekler A Utikal JS Grabe N SChadendorf D Klode J . Skin cancer classification using convolutional neural networks: Systematic review. J Med Internet Res (2018) 20:e11936. doi: 10.2196/11936 30333097 PMC6231861

[19] Nasr-Esfahani E Samavi S Karimi N Soroushmehr SM Jafari MH Ward K . Melanoma detection by analysis of clinical images using convolutional neural network. Annu Int Conf IEEE Eng Med Biol Soc (2016) 2016:1373–6. doi: 10.1109/EMBC.2016.7590963 28268581

[20] Murugan A Nair SAH Preethi AAP Kumar KPS . Diagnosis of skin cancer using machine learning techniques. Microprocess Microsyst (2021) 81:103727. doi: 10.1016/j.micpro.2020.103727

[21] Damiani G Grossi E Berti E Conic RRZ Radhakrishna U Pacifico A . Artificial neural networks allow response prediction in squamous cell carcinoma of the scalp treated with radiotherapy. J Eur Acad Dermatol Venereol (2020) 34:1369–73. doi: 10.1111/jdv.16210 31968143

[22] Pranckutė R . Web of science (WoS) and scopus: the titans of bibliographic information in today’s academic world. Publications. Web Science (2021) 9:12. doi: 10.3390/publications9010012

[23] Chen CM Leydesdorff L . Patterns of connections and movements in dual-map overlays: A new method of publication portfolio analysis. J Assn Inf Sci Tec (2014) 65:334–51. doi: 10.1002/asi.22968

[24] Sauter D Lodde G Nensa F SChadendorf D Livingstone E Kukuk M . Deep learning in computational dermatopathology of melanoma: A technical systematic literature review. Comput Biol Med (2023) 163:107083. doi: 10.1016/j.compbiomed.2023.107083 37315382

[25] Melarkode N Srinivasan K Qaisar SM Plawiak P . AI-powered diagnosis of skin cancer: A contemporary review, open challenges and future research directions. Cancers (Basel) (2023) 15:1183. doi: 10.3390/cancers15041183 36831525 PMC9953963

[26] Samuel AL . Machine learning. Technol Rev (1959) 62:42–5.

[27] Lu H Han T Li F Yang J Hou Z . Global trends and hotspots in research of robotic surgery in oncology: A bibliometric and visual analysis from 2002 to 2021. Front Oncol (2022) 12:1055118. doi: 10.3389/fonc.2022.1055118 36439475 PMC9691977

[28] Wang H Shi J Shi S Bo R Zhang X Hu Y . Bibliometric analysis on the progress of chronic heart failure. Curr Probl Cardiol (2022) 47:101213. doi: 10.1016/j.cpcardiol.2022.101213 35525461

[29] Sridharan B Sharma AK Lim HG . The role of ultrasound in cancer and cancer-related pain-A bibliometric analysis and future perspectives. Sensors (Basel) (2023) 23:7290. doi: 10.3390/s23167290 37631826 PMC10458834

[30] Cheng L Liu Y Ma Q Yan S Li H Zhan H . Bibliometric analysis of the global publication activity in the field of relapsing polychondritis during 1960–2023. Clin Rheumatol (2023). doi: 10.1007/s10067-023-06741-2 37620677

[31] Hamet P Tremblay J . Artificial intelligence in medicine. Metabolism (2017) 69S:S36–40. doi: 10.1016/j.metabol.2017.01.011 28126242

[32] SChadendorf D van Akkooi ACJ Berking C Griewank KG Gutzmer R Hauschild A . Melanoma. Lancet (2018) 392:971–84. doi: 10.1016/S0140-6736(18)31559-9 30238891

[33] Pai VV Pai RB . Artificial intelligence in dermatology and healthcare: An overview. Indian J Dermatol Venereol Leprol (2021) 87:457–67. doi: 10.25259/IJDVL_518_19 34114421

[34] Russakovsky O Deng J Su H Krause J Satheesh S Ma S . ImageNet large scale visual recognition challenge. Int J Comput Vis (2015) 115:211–52. doi: 10.1007/s11263-015-0816-y

[35] LeCun Y Bengio Y Hinton G . Deep learning. Nature (2015) 521:436–44. doi: 10.1038/nature14539 26017442

[36] Esteva A Kuprel B Novoa RA Ko J Swetter SM Blau HM . Dermatologist-level classification of skin cancer with deep neural networks. Nature (2017) 542:115–8. doi: 10.1038/nature21056 PMC8382232 28117445

[37] Lallas A Tzellos T Kyrgidis A Apalla Z Zalaudek I Karatolias A . Accuracy of dermoscopic criteria for discriminating superficial from other subtypes of basal cell carcinoma. J Am Acad Dermatol (2014) 70:303–11. doi: 10.1016/j.jaad.2013.10.003 24268311

[38] Mobiny A Singh A Van Nguyen H . Risk-aware machine learning classifier for skin lesion diagnosis. J Clin Med (2019) 8:1241. doi: 10.3390/jcm8081241 31426482 PMC6723257

[39] Marchetti MA Codella NCF Dusza SW Gutman DA Helba B Kalloo A . Results of the 2016 International Skin Imaging Collaboration International Symposium on Biomedical Imaging challenge: Comparison of the accuracy of computer algorithms to dermatologists for the diagnosis of melanoma from dermoscopic images. J Am Acad Dermatol (2018) 78:270–277.e1. doi: 10.1016/j.jaad.2017.08.016 28969863 PMC5768444

[40] UdriŞtoiu AL Stanca AE Ghenea AE Vasile CM Popescu M UdriŞtoiu ŞC . Skin diseases classification using deep leaning methods. Curr Health Sci J (2020) 46(2):136–40. doi: 10.12865/CHSJ.46.02.06 PMC7445643 32874685

[41] Brinker TJ Hekler A Enk AH Berking C Haferkamp S Hauschild A . Deep neural networks are superior to dermatologists in melanoma image classification. Eur J Cancer (2019) 119:11–7. doi: 10.1016/j.ejca.2019.05.023 31401469

[42] Wu H Chen H Wang X Yu L Yu Z Shi Z . Development and validation of an artificial intelligence-based image classification method for pathological diagnosis in patients with extramammary Paget’s disease. Front Oncol (2021) 11:810909. doi: 10.3389/fonc.2021.810909 35118000 PMC8804211

[43] Andrew TW Hamnett N Roy I Garioch J Nobes J Moncrieff MD . Machine-learning algorithm to predict multidisciplinary team treatment recommendations in the management of basal cell carcinoma. Br J Cancer (2022) 126:562–8. doi: 10.1038/s41416-021-01506-7 PMC8854628 34471257

[44] Sohn W Finley DS Jakowatz J Ornstein DK . Robot-assisted laparoscopic transperitoneal pelvic lymphadenectomy and metastasectomy for melanoma: Initial report of two cases. J Robot Surg (2010) 4:129–32. doi: 10.1007/s11701-010-0189-8 PMC2917550 20730107

[45] Kim SI Lee S Jeong CW Kim HS . Robot-assisted anterior pelvic exenteration in vulvovaginal Malignant melanoma. Gynecol Oncol (2018) 148:430–1. doi: 10.1016/j.ygyno.2017.12.022 29276058

[46] Sánchez A Sotelo R Rodriguez O Sánchez R Rosciano J Medina L . Robot-assisted video endoscopic inguinal lymphadenectomy for melanoma. J Robot Surg (2016) 10:369–72. doi: 10.1007/s11701-016-0599-3 27173971

[47] Giansanti D . The artificial intelligence in teledermatology: A narrative review on opportunities, perspectives, and bottlenecks. Int J Environ Res Public Health (2023) 20:5810. doi: 10.3390/ijerph20105810 37239537 PMC10217851

[48] Ho D Quake SR McCabe ERB Chng WJ Chow EK Ding X . Enabling technologies for personalized and precision medicine. Trends Biotechnol (2020) 38:497–518. doi: 10.1016/j.tibtech.2019.12.021 31980301 PMC7924935

[49] Schork NJ . Artificial intelligence and personalized medicine. Cancer Treat Res (2019) 178:265–83. doi: 10.1007/978-3-030-16391-4_11 PMC7580505 31209850

[50] Hekler A Utikal JS Enk AH Hauschild A Weichenthal M Maron RC . Superior skin cancer classification by the combination of human and artificial intelligence. Eur J Cancer (2019) 120:114–21. doi: 10.1016/j.ejca.2019.07.019 31518967

[51] Yang J Wu X Liang J Sun X Cheng MM Rosin PL . Self-paced balance learning for clinical skin disease recognition. I.E.E.E. Trans Neural Netw Learn Syst (2020) 31:2832–46. doi: 10.1109/TNNLS.2019.2917524 31199274

[52] Tobore I Li J Yuhang L Al-Handarish Y Kandwal A Nie Z . Deep learning intervention for health care challenges: Some biomedical domain considerations. J.M.I.R. MHealth UHealth (2019) 7:e11966. doi: 10.2196/11966 PMC6696854 31376272

